# Community-based mental health interventions for reducing youth suicidal thoughts and behaviours: a systematic review and meta-analysis

**DOI:** 10.1186/s12888-025-07527-3

**Published:** 2025-10-30

**Authors:** Lulu Barker, Samantha Sijing Wang, Md Nazmul Huda, James Rufus John, Weng Tong Wu, Ping-I Daniel Lin, Valsamma Eapen

**Affiliations:** 1 Discipline of Psychiatry and Mental Health, School of Clinical Medicine, University of New South Wales, Sydney, NSW 2052 Australia; 2 Ingham Institute of Applied Medical Research and Liverpool Hospital, Sydney, NSW Australia; 3 Academic Unit of Infant, Child and Adolescent Psychiatric Services, South Western Sydney Local Health District, Sydney, Australia; 4 Department of Psychiatry and Behavioral Neuroscience, Saint Louis University School of Medicine, Missouri, USA

**Keywords:** Community-based intervention, Suicide prevention, Adolescents, Suicidal ideation, Suicide attempt, Suicide planning, Systematic review, Non-suicidal self-injury, Mental health programs

## Abstract

**Background:**

Rates of suicidal thoughts and behaviours (STB) and non-suicidal self-injury (NSSI) in children and young people (CYP) are on the rise globally. Community-based mental health interventions may offer accessible and scalable support, however there is a paucity of evidence on their effectiveness in this population. We aim to provide an up-to-date synthesis and evaluation of evidence on whether community-based mental health programs targeting CYP (< 18 years) reduce STB and NSSI.

**Methods:**

Four electronic databases (PsycINFO, EMBASE, PubMed, and CINAHL) and Google Scholar were searched from October 2013 to June 2024. Eligible studies included controlled trials of CYP (sample mean age < 18 years) conducted in community settings, examining at least one primary outcome of STB or NSSI. Methodological quality was assessed, and findings were summarised using narrative synthesis and, where appropriate, quantitatively through meta-analyses.

**Results:**

From 1553 records, 13 studies met inclusion criteria, spanning universal or targeted school-based, digital, family-focused, and community-based programs across 12 countries. Meta-analysis revealed significant reduction in suicidal ideation (Pooled OR = 0.57, 95% CI: 0.44–0.72, *p* < .00001) and suicide attempts (OR = 0.43, 95% CI: 0.24–0.76, *p* = .004) following intervention. Suicide planning and NSSI outcomes were less conclusive due to limited data and moderate to high heterogeneity.

**Conclusions:**

Community-based interventions are effective in reducing suicidal ideation and suicide attempts among CYP, however their effectiveness at reducing NSSI and suicide planning is unclear. These results support the integration of community-based suicide prevention programs into national mental health strategies. Further rigorous, long-term, and culturally inclusive research is necessary to inform efficacious and scalable suicide prevention strategies.

**Systematic review registration:**

PROSPERO CRD42024501756.

**Clinical trial number:**

Not applicable.

## Background

Suicide and non-suicidal self-injury (NSSI) continue to pose a significant global health threat for children and young people (CYP), with incidence rates on the rise globally [1–4]. While studies in high-income countries (HICs) reveal an increasing prevalence of NSSI among younger aged groups [5, 6], recent data highlights an alarming burden in low- and middle-income countries (LMICs), where, concerningly, mental health services remain limited [7]. In a 2015 survey of over 220,000 adolescents from LMICs, 16.9% of adolescents reported suicidal ideation, 17.0% had made a suicide plan, and 17.0% had attempted suicide in the preceding 12-month period [8]. More recent multi-country syntheses since 2020 also document substantial adolescent suicidal ideation and attempts across LMICs, underscoring persistent global need for accessible intervention [9, 10]. In the United States, the Centers for Disease Prevention and Control’s 2013–2023 Youth Risk Behaviour Survey (YRBS) similarly reports persistently high levels of suicidal thoughts and behaviours, with marked disparities by sex and LGBTQ + status [11]. Globally, both suicidal ideation and NSSI function as predictive risk factors for future suicide attempts, with 17% of pre-adolescents experiencing suicidal ideation progressing to an attempt [12, 13]. In addition, rates of suicide attempt, suicidal ideation and NSSI in CYP, notably in girls, rose during the COVID-19 pandemic [14], with inpatient admissions related to deliberate NSSI increasing by 82% in Australia [15]. While there was a slight decline following easing restrictions, NSSI numbers remain higher than the pre-COVID-19 level [15]. Even so, the full burden of suicidal thoughts and behaviours (STB), specifically suicidal ideation, suicide planning, suicide attempt and NSSI is likely underestimated, due to the misclassification and underreporting in clinical, administrative, and survey data, as well as uncounted psychological sequelae for families and communities (grief, post-traumatic stress, complex bereavement) [16, 17]. Thus, effective and accessible interventions to reduce STB in CYP are urgently needed.

STB frequently begin in adolescence, but stigma and limited mental health literacy often delay necessary help-seeking by CYP [18]. This delay has serious repercussions, contributing to a triple burden encompassing health, psychosocial and economic domains [16, 17]. From a health perspective, STB can amplify psychopathological symptoms, which in turn can further aggravate STB [19]. Psychosocially, STB disrupts essential developmental milestones, undermines peer and family relationships, and negatively affects long-term quality of life [20]. In economic terms, both direct healthcare costs (e.g., hospitalisations, psychiatric care) and indirect losses (e.g., productivity deficits, caregiving burdens) place a considerable strain on families and healthcare systems [21, 22]. Together, these factors underscore the need for effective and accessible interventions addressing STB early in adolescence.

Internationally, various strategies addressing STB and NSSI have been implemented in an effort to alleviate this burden [23, 24]. However, despite these initiatives, gaps in accessibility, engagement, and long-term effectiveness persist. For example, emergency departments (EDs) are often the first point of contact for CYP in acute distress. However, EDs are frequently overcrowded, with significant shortage of mental health specialists, and hence may not offer the follow-up care required to prevent further NSSI or suicide attempts [25]. Clinical mental health services may face resource and capacity limitations, subjecting CYP to long wait times and high costs [26–28]. Non-profit organisations providing mental health services, such as Headspace (Australia’s primary national youth mental health service offering assessment, brief psychological therapies, and care navigation for adolescents aged between 12 and 25) and similar services internationally, are challenged by inconsistent availability in rural areas and limited capacity to meet demand, especially for moderate to severe presentations [29]. These barriers, alongside the increasing rates of STB and NSSI among CYP, underscore the crucial need for more accessible and sustainable interventions.

Community-based interventions have emerged as a promising solution to address the gaps in accessibility, engagement, and long-term effectiveness present in traditional and clinical mental health services. Defined as programs, strategies or initiatives which take place in various community settings (e.g., schools, correction centres, community centres etc.), these interventions integrate support into familiar environments, aiming to reduce mental health disparities and optimise the psychological wellbeing of target groups before crises escalate to the point of requiring emergency care [30, 31]. Evidence highlights the effectiveness of school-based interventions, community-wide initiatives, peer support programs, and brief contact interventions, in reducing STB [32–35]. By embedding mental health support within accessible community spaces, these interventions reduce reliance on overburdened emergency departments, mitigate barriers such as transportation and financial costs, and ensure support reaches marginalised groups who may otherwise avoid formal healthcare services. As community-based interventions continue to expand, they provide an effective, scalable, and sustainable approach to improving mental health outcomes for CYP globally.

Although certain community-based programs have shown promise [36–39], their effectiveness among CYP remains elusive. Past reviews have either focused only on school-based interventions [40–42], included a wide range of clinical and pharmacological approaches alongside community-based strategies [43–46], or emphasised outcomes such as help-seeking over STB [47, 48]. Consequently, the unique impact of interventions across any community setting (including, but not restricted to, school-based interventions), independent of clinical or pharmacological interventions, in preventing STB among youth has not been comprehensively evaluated. To address this evidence gap, this systematic review aims to identify and map the breadth of community-based mental health programs for CYP and evaluate their efficacy in reducing STB. We hope that the findings of this review will guide resource allocation and help inform future preventive strategies capable of delivering meaningful, long-term benefits to at-risk children and adolescents.

## Methods

This systematic review was conducted following the Preferred Reporting Items for Systematic Reviews and Meta-Analyses (PRISMA) guidelines and registered with the University of York Centre for Reviews and Dissemination (PROSPERO: registration number CRD42024501756).

### Search strategy

Four electronic databases (PsycINFO, EMBASE, PubMed, and CINAHL) were searched for peer-reviewed articles published from October 2013 to June 2024. These databases were selected to capture a broad range of empirical studies in psychology, medicine, nursing, and allied health. In addition, Google Scholar was searched using relevant Boolean keyword combinations for articles published in English, and 2013–2024. The first ~ 200 results (≈ 20 pages) were screened by relevance for each core query. Duplicates were removed before screening. This was further supplemented by hand-searched reference lists of relevant systematic reviews. Inclusion of studies was restricted to 2013–2024 to reflect a period of definitional consolidation and increased measurement consistency in suicidology, characterised by the Centers for Disease Control and Prevention (CDC)’s uniform self-directed violence definitions [49], Diagnostic and Statistical Manual of Mental Disorders – Fifth Edition (DSM-5)’s distinction between NSSI and STB [50–53], and widespread uptake of standardised assessment tools (e.g., The Columbia-Suicide Severity Rating Scale, C-SRSS) [54–56]. Limiting to this modern era reduces cross-study variability stemming from older, heterogenous terms (e.g., ‘parasuicide’, broad ‘self-harm’ irrespective of intent) and enhances comparability of outcomes such as ideation, planning, attempts, and NSSI. Table 1 presents the search terms used in this review.

**Table 1:** Search terms used in this review

PICOS	Search terms
Patient, Population or Problem	Adolescen* OR child* OR young people OR teen* OR minor* OR juvenile* young person* OR patient* OR youth OR young individual* OR young population OR suicide attempter*
Interventions and Comparisons	Community mental health OR community care OR community-based care OR community health service OR intervention* OR treatment* OR recovery OR prevention intervention* OR program*
Outcomes	Suicide OR suicide attempt* OR, cutting OR head-banging OR suicide ideation OR suicidal thought* OR suicidal behavio* OR self-injur* OR self-harm OR self-destructive behavio* OR program* OR program effectiveness OR program efficacy OR service* OR service efficacy OR intervention efficacy OR service effectiveness OR suicidality OR suicidality reduction*
Study Design	Clinical trial* OR Randomised controlled trial* OR Cluster randomised trial* OR quasi experimental* OR RCT

### Inclusion and exclusion criteria

This review included articles that met the following criteria: (1) interventions focused primarily on STB, specifically suicidal ideation, suicide planning and suicide attempt, and NSSI as a main outcomes; (2) conducted in a community-based settings (e.g., schools, community centres, or web-based programs); (3) sample mean age was under 18 years of age; (4) used a controlled design, such as a randomised controlled trial (RCT) or controlled trials using alternative treatment group allocation methods; and (5) were published in English. We required STB/NSSI to be a pre-specified primary outcome to align with trial design and evidence-synthesis standards: primary outcomes are typically used for sample-size determination and analytic hierarchy, whereas secondary outcomes are often exploratory, underpowered, vulnerable to selective reporting, and therefore, contribute greater to indirectness and imprecision in pooled estimates [57–61]. As randomisation may be impractical or ethically challenging in some community settings, in order to capture a more comprehensive range of intervention research, both randomised and non-randomised designs with a control group were included.

Articles were excluded if: (1) the intervention was delivered in a clinical setting (rather than a community setting); (2) the primary outcome was not a direct component of STB (i.e., suicidal ideation, suicide planning, suicide attempt or NSSI); (3) the study design was other than an RCT or non-randomised controlled-trial study (such as conference abstracts, reviews, case reports, opinion pieces, protocols); (4) the sample mean age was over 18 years; (5) was not published in English.

### Screening and selection process

The retrieved records from each database were uploaded onto Rayyan, an artificial intelligence-powered tool for systematic review management [62]. Two reviewers (SSW and WTW) independently screened the titles and abstracts of articles for eligibility before conducting the full-text screening, remaining blind to the other’s decisions throughout the process. Discrepancies were then resolved through discussion with a third reviewer (MNH).

### Data extraction

Data extraction was performed by three reviewers (WTW, SSW, and LB) using Microsoft Excel spreadsheet software (Microsoft Corporation). Extracted data included author, publication year, country of setting, sample size, participant information (i.e., age range, gender distribution), primary outcomes (i.e., suicidal ideation, suicide attempts, suicide planning, and NSSI), presence and duration of a follow-up, key findings (significance and effect size) of the outcomes, a description of the intervention, the effectiveness of the intervention, and the quality appraisal score.

### Quality assessment and risk of bias

The methodological validity of each of the included studies was independently evaluated by two reviewers (LB and MNH) using the Joanna Briggs Institute (JBI) critical appraisal checklists for RCTs, and the JBI critical appraisal checklists for quasi-experimental studies, both of which offer robust criteria for assessing study quality [63, 64]. Based on summary scores, the quality of studies was rated as good (≥ 8 out of 13 ‘yes’ answers when using the RCT checklist, or ≥ 6 out of 9 ‘yes’ answers when using the quasi-experimental checklist), fair (6–7 of 13, or 4–5 of 9), or poor (≤ 5 of 13, or ≤ 3 of 9). In addition, the overall strength of the evidence was evaluated using the Grading of Recommendations Assessment, Development and Evaluation (GRADE) criteria [65], which considers risk of bias, inconsistency, indirectness, imprecision, and publication bias. Quality ratings were independently assigned by LB and SSW, with any discrepancies resolved through discussion.

### Data synthesis and analysis

An overall data synthesis was conducted using narrative synthesis to capture the breadth of findings, given the heterogeneity of outcomes and interventions. Meta-analyses were conducted for outcomes reported as proportions in each group or as odds ratios (ORs) to synthesise effect estimates across studies. For dichotomous outcomes (e.g., clinically significant presence vs. absence of suicidal ideation), ORs with corresponding 95% confidence intervals (CIs) were computed and presented as forest plots. Given the anticipated heterogeneity across study populations, settings, and intervention modalities, a random-effects model was implemented using the inverse-variance weighting method. This approach accounts for both within-study and between-study variance, thereby enhancing the generalisability of the pooled estimates [66]. Statistical heterogeneity was assessed using the I² statistic [66], of which the values >50% were interpreted as indicative of substantial heterogeneity. All meta-analyses were performed using Review Manager (RevMan) version 5.4, following Cochrane guidelines.

## Results

### Search results

**Fig. 1:**
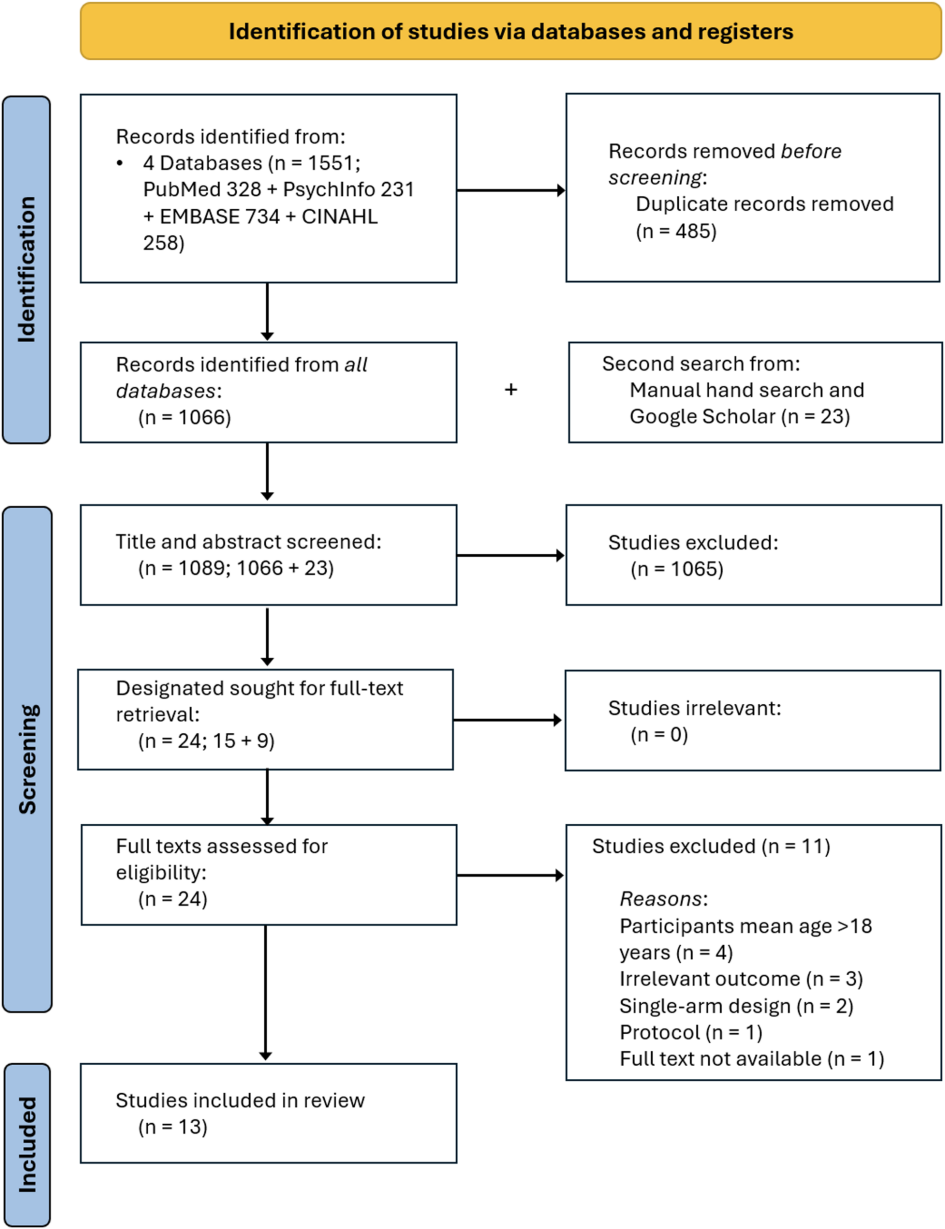
Study selection process (PRISMA-P)

Searches of the electronic databases resulted in 1551 articles for consideration, of which 1066 remained after removing duplicates. A further 21 articles were identified from searching Google Scholar, leaving 1087 to undergo title and abstract screening. From this process, 22 articles were deemed eligible for full-text screening. Reasons for exclusions were: (1) sample mean age over 18 years of age (*n* = 4), (2) primary outcome/s were other than NSSI or STB (*n* = 3), (3) study utilised a single-arm design (*n* = 2), (4) study was a protocol (*n* = 1) and (5) full text not available (*n* = 1). A final 13 studies remained after full-text screening [67–79]. A flowchart of the selection process was produced according to PRISMA guidelines [80] (see Fig. 1).

### Characteristics of included studies

Table 2 presents the characteristics of included studies, which were conducted across 12 countries (United States of America = 9, Austria = 1, Australia, = 2, multinational [Austria, Estonia, France, Germany, Hungary, Ireland, Italy, Romania, Slovenia and Spain] = 1). Of the 13 studies, 12 were RCTs [67–78] and one (Schilling et al., [79]) was a non-randomised controlled trial. Together, these studies included an overall sample size of 18,128 study participants, aged between 11 and 19 years of age with varying gender distribution across studies.

**Table 2:** Characteristics of included studies

Study	Country	Participants (Age, gender distribution)	Relevant STB/NSSI outcome	Intervention location and characteristics	Intervention description	Follow-up	Quality assessment score
Braun et al. (2023) [76]	Austria	*N* = 299 Female (72.25%) and male (27.75%) youth, aged 14–19 years old, mean age (SD) = 17.95 years (1.19). 67.56% of Austrian nationality.	Suicidal ideation	Community centres with digital component, universal	Suicide prevention videos developed by adolescents for adolescents which depicted a relatable protagonist engaging in help-seeking behaviours to decrease STB (program unnamed)	4 weeks	Good (12/13) þ
Dobias et al. (2021) [77]	USA	*N* = 565 Gender-diverse sample, majority identifying as female (67%) and male (8–9%), mean age (SD) = 14.94 (0.98)	Self-injurious behaviour	Web-based, indicated	Project SAVE (Stop Adolescent Violence Everywhere). A single-session, web-based intervention aiming to decrease the rate of NSSI through teaching adolescents cognitive behavioural therapy techniques.	3 months	Good (12/13) þ
Grummit et al. (2022) [72]	Australia	*N* = 1636 Female (40%) and male (60%) students, mean age (SD) = 13.3 years (0.5)	Suicidal ideation	School setting, clustered at school-level, selective	Preventure. Targeted adolescents with personality traits associated with an increased likelihood of substance abuse and hopelessness.	3 years	Good (9/13) þ
Hetrick et al. (2017) [78]	Australia	*N* = 50 Female (82%), 8th to 12th graders.	Suicidal ideation	Web-based, selective	Reframe-IT. Web-based cognitive behavioural therapy for adolescents experiencing STB.	12 weeks	Good (9/13) þ
Kerr et al. (2014) [74]	USA	*N* = 166 Female (100%), Mean age (SD) = 15.30 (1.17)	Suicidal ideation, suicide attempt	Multidimensional (school setting and in foster care), selective	Multidimensional Treatment Foster Care. Placed female youth with high rates of delinquency from out-of-home care into homes with foster parents trained to deliver a therapeutic behavioural program.	Person specific. For Cohort 1, follow-up occurred at 3, 6, 12, 15, 18, 30, and 36 months, then again at long-term (mean = 9.81 years), and 6, 12, 18, and 24 months after this. Cohort 2 was assessed at 3, 12, and 24 months, and at long-term follow-up (mean = 4.69 years post-baseline), then 6, 12, 18, and 24 months after this.	Good (12/13) þ
Petrova et al. (2015) [68]	USA	*N* = 706 Female (49.2%) and male (50.8%), 9th to 12th graders.	Suicidal ideation	School setting, clustered at school-level, universal	Sources of Strength. Aimed to promote social connectedness and help-seeking behaviours through the use of ‘peer leaders’; other peers trained to deliver suicide-prevention sessions and messaging in school.	No follow-up	Good (8/13) þ
Robinson et al. (2016) [71]	USA	*N* = 758 Female (60%) and male (40%), age range 14–18 years (Mean and SD not calculable due to reporting)	Suicidal ideation	School setting, clustered at school-level, universal	Adapted-Coping with Stress course. ACW-S is tailored to reduce STB specifically in African-American adolescents through improving adaptive coping capabilities.	No follow-up	Fair (7/13) þ
Robinson et al. (2024) [70]	USA	*N* = 410 Female (56%) and male (44%), mean age (SD) = 14.5 years (0.59)	Suicidal ideation	School setting, clustered at school-level, universal	Adapted-Coping with Stress course. ACW-S is tailored to reduce STB specifically in African-American adolescents through improving adaptive coping capabilities.	6 months, 12 months	Good (9/13) þ
Sandler et al. (2016) [73]	USA	*N* = 244 Gender distribution not reported. Mean age (SD) = 11.39 years (2.43)	Suicidal ideation, suicide attempt	Multidimensional (Assessments occurred at home, intervention sessions occurred in community settings; further information not revealed), Selective and upstream	Family Bereavement Program. An upstream intervention targeting youth experiencing parental bereavement.	6 years, 15 years	Good (9/13) þ
Schilling et al. (2014) [79]	USA	*N* = 386 Female (52.6%) and male (47.4%) 5th to 8th graders.	Suicidal ideation, suicide attempt, suicide planning	School setting, clustered at school-level, universal	Signs of Suicide. Aimed to increase awareness and understanding of depressive symptomology to improve help-seeking behaviours.	3 months	Good (6/9)*
Schilling et al. (2016) [67]	USA	*N* = 1052 Female (49.2%) and male (58%) 9th graders.	Suicidal ideation, suicide attempt, suicide planning	School setting, clustered at school-level, universal	Signs of Suicide	3 months	Good (8/13) þ
Vidot et al. (2016) [75]	USA	*N* = 746 Female (48%) and male (52%), mean age (SD) = 13.9 years (0.67)	Suicidal ideation, suicide attempt	Intervention occurred in community settings; further information not revealed, selective	Familias Unidas. A family-focused intervention for Hispanic youth and their primary caregiver/s, which aimed to improve overall family functioning.	6 months, 18 months, 30 months	Good (8/13) þ
Wasserman et al. (2015) [69]	International (Europe)	*N* = 11,110 Female (59%) and male (41%), mean age (SD) = 14.80 years (0.8)	Suicidal ideation, suicide attempt	School setting, clustered at school-level, universal, selective and indicated	Study compared three interventions: (1) Question, Persuade, and Refer. Aimed to improve teachers’ ability to recognise suicide risk in their students and coach them on how to instigate effective communication with at-risk students. (2) The Youth Aware of Mental Health Program. Aimed to improve student’s adaptive coping capabilities and awareness of suicide risk factors (3) ProfScreen. Students whose survey answers were flagged as moderate or high risk were put into contact with mental health professionals.	3 months, 12 months	Good (9/13) þ

þ Scored using The JBI Critical Appraisal Checklist for RCTs *Scored using The JBI Critical Appraisal Checklist for Quasi-Experimental Studies

### Characteristics of community-based interventions

**Fig. 2:**
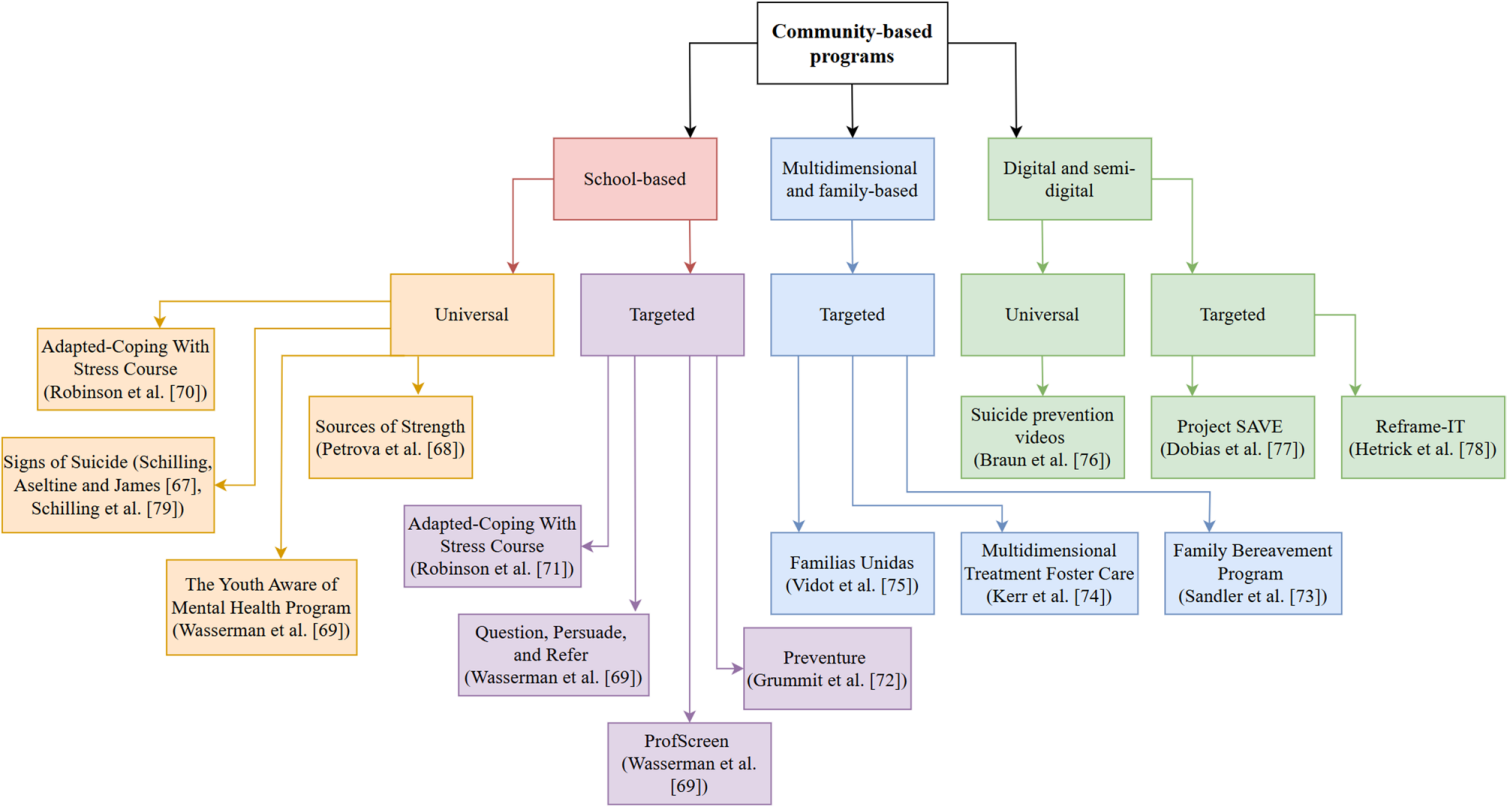
Flowchart of included interventions

The 13 included studies have been organised by modality in order to highlight the diversity of strategies employed (see Fig. 2).

#### School-based interventions

##### Universal programs

Five of the 13 studies evaluated universal school-based interventions, all of which were clustered at the school-level [67–70, 79] and delivered to all students irrespective of their risk status. Of these five studies, two programs aimed to improve help-seeking behaviours, albeit through differing methods [67, 68, 79]. Signs of Suicide, evaluated by both Schilling, Aseltine and James [67] and Schilling et al., [79] encouraged students to help-seek through promoting awareness of and familiarity with depressive symptomology (although conducted by the same author, Schilling, Aseltine and James [67] utilised a novel sample, and so both studies were included in the review). Petrova et al., [68] assessed Sources of Strength, which is designed to increase help-seeking behaviours by promoting social-connectedness with ‘peer leaders’: other peers trained to deliver suicide-prevention sessions and messaging in school. Wasserman et al., [69] and Robinson et al., [70] both examined universal programs intended to improve student’s adaptive coping abilities via the Youth Aware of Mental Health Program [YAM] and the Adapted-Coping with Stress course [ACW-S], respectively. However, YAM also aims to improve awareness of suicide risk factors, and the A-CWS is culturally tailored specifically for African American adolescents. The duration of follow-up varied from no follow-up [68] to 12-months [69], with an average follow-up time of 6-months.

##### Targeted (Selective and indicated) programs

Another three studies reported on targeted school-based interventions [69, 71, 72]. Unlike the universal approach, the selective or indicated programs are targeted interventions for subgroups or individuals identified as at higher risk for STB or NSSI. Robinson et al., [71], also examined the A-CWS intervention, but utilised a novel cohort (as compared to the subsequent Robinson et al., [70]) and excluded participants who did not evidence some risk when screened for STBs prior to the intervention. No follow-up was reported. Grummitt et al., [72] examined Preventure, a program targeting adolescents with personality traits associated with an increased likelihood of substance abuse and hopelessness. Follow-up was conducted at 3-years. Similarly, ProfScreen, examined by Wasserman et al., [69], targeted students whose survey answers indicated they were at risk for STB. Wasserman et al., [69] also examined Question, Persuade, and Refer (QPR), a gate-keeper training program. Follow-up was administered at 3- and 12-months for both ProfScreen and QPR [69].

#### Multidimensional and family-focused interventions

Multidimensional and family-focused interventions were delivered in settings such as community centres or family homes, often targeting complex, multidimensional risk factors [73–75]. Both Sandler et al., [73] and Kerr et al., [74] assessed complex risk factors and, alongside Vidot et al., [75], were categorised as selective programs. Sandler et al., [73] examined the Family Bereavement Program (FBP), an upstream intervention targeting youth experiencing parental bereavement. Participants were recruited through schools and media presentations, assessments occurred in the home setting, and intervention sessions were conducted in community settings, details of which were not specified. As the FBP is upstream, follow-up occurred six- and 15-years post intervention. Kerr et al. [74] evaluated Multidimensional Treatment Foster Care, which targeted female youth with high rates of delinquency in out-of-home care. Participants were placed in homes with foster parents trained to deliver a therapeutic behavioural program across school and home settings. Additionally, participants were also divided into two cohorts, where the second received additional psychoeducational modules focussing on risky sexual behaviour and substance abuse alongside the base program. Follow-up timing varied, but was administered, on average, at 8.8 years [74]. Finally, Vidot et al., [75] examined Familias Unidas, a selective, family-focused intervention for Hispanic youth and their primary caregiver/s which aimed to improve overall family functioning. The recruitment method and session locations were not reported. Follow-up was conducted at 6-, 18- and 30-months post baseline.

#### Digital and semi-digital interventions

Three studies leveraged scalable and flexible formats to deliver mental health support via digital platforms [76–78]. Braun et al., [76] examined the effectiveness of adolescent-produced videos depicting a youth engaging in help-seeking behaviours when facing STB. These videos were shown in community centres across Austria. Dobias et al., [77] examined Project SAVE (Stop Adolescent Violence Everywhere), a single-session, web-based intervention aiming to decrease the rate of NSSI through cognitive behavioural therapy techniques. Hetrick et al., [78] assessed Reframe-IT, a web-based cognitive behavioural therapy for adolescents experiencing STB. Although Hetrick et al., [78] did recruit participants from schools, as the intervention was delivered entirely online (through modules consisting of videos and a message board where participants could communicate with a clinical psychologist), we have categorised the intervention as web-based. Braun et al., [76] conducted follow-up at 4-weeks, while both Dobias et al., [77] and Hetrick [78] conducted follow-up at 3-months.

### Effectiveness of community-based interventions on suicidal behaviours

Four primary outcomes were assessed across the included studies: suicidal ideation, suicide attempts, suicide planning and NSSI. While six studies provided quantitative data suitable for meta-analysis, findings of the other five studies were summarised using narrative synthesis.

#### Suicidal ideation

**Fig. 3:**
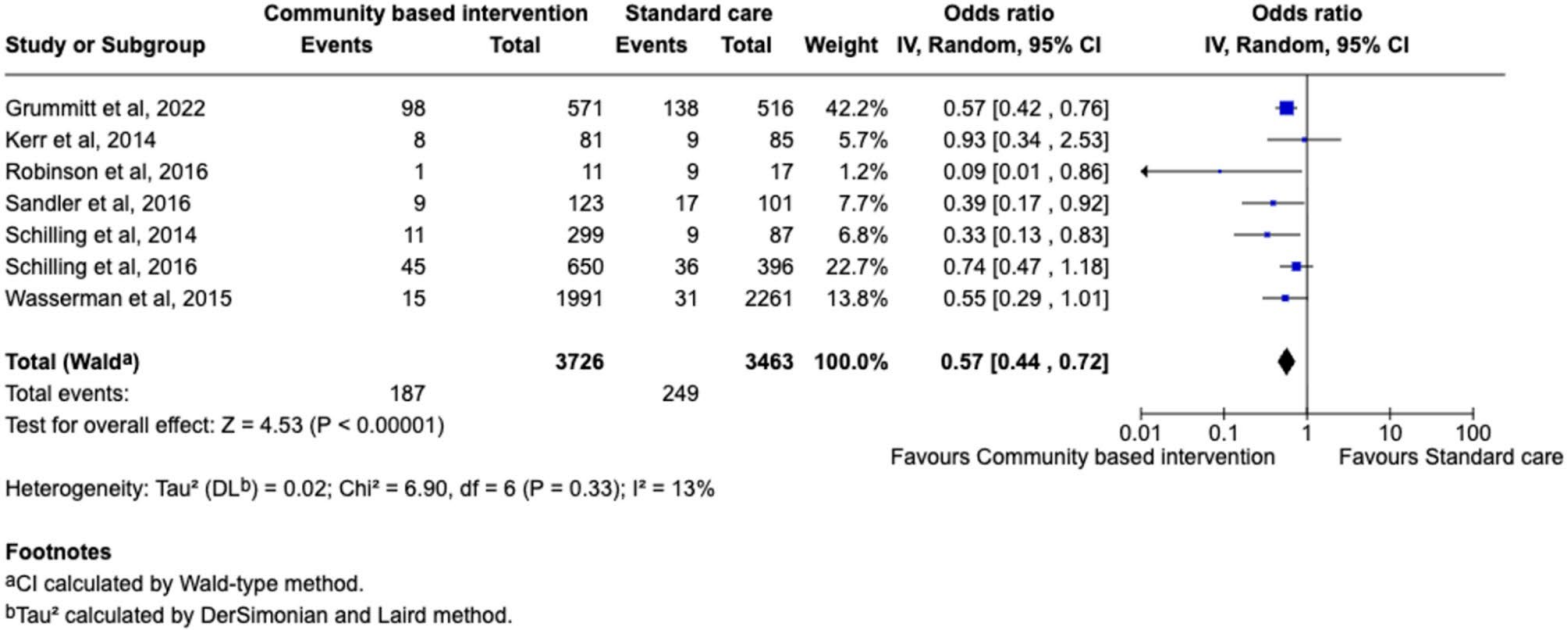
Forest plot of the efficacy of community-based intervention (versus standard care) on suicidal ideation outcome

A total of twelve studies [67–76, 78, 79] reported on the impact of community-based interventions to reduce suicidal ideation, seven of which had sufficient data for inclusion in the meta-analysis [67, 69, 71–74, 79]. The meta-analysis demonstrated a significant improvement in suicidal ideation following community-based intervention (Pooled OR = 0.57, 95% CI: 0.44–0.72, *p* < .00001) with 43% reduction in risk (see Fig. 3). Low heterogeneity indicated consistent effect sizes across studies (*I²*=13%). Further, when narratively synthesised, findings of the twelve studies continued to indicate a promising trend [67–76, 78, 79]. Sandler et al., [73], Robinson et al., [71] and Grummit et al., [72] reported significant reductions in ideation following intervention, with Grummit et al., [72] observing a 20% decrease in suicidal ideation per year, for three years. Kerr et al., [74] reported a marginally significant reduction of suicidal ideation in the first cohort, and a significant reduction in suicidal ideation in the second. Although Petrova et al., [68] reported a significant reduction in suicidal ideation, no follow-up was conducted to assess if these results maintained over time. Finally, only one of the three interventions assessed by Wasserman et al., [69] evidenced a significant reduction in suicidal ideation (YAM). The other five studies showed promising, but not significant, results. Both Schilling et al. [79] and Schilling, Aseltine and James [67] found a significant reduction in suicidal ideation, suicide attempt and suicide planning when assessed as a composite outcome (suicidal behaviours), however ideation was not significant as an individual outcome in either study. Robinson et al., [70] did not find evidence to suggest A-CWS was effective as a universal treatment but did observe that A-CWS significantly reduced ideation in participants with higher suicidal ideation at baseline, suggesting potential to be effective as an indicated intervention. These findings align with Robinson et al., [71], who reported significant reductions in suicidal ideation following A-CWS as a targeted intervention. However, it must be taken into consideration that Robinson et al., [70] conducted follow-up where Robinson et al., [71] did not. Vidot et al., [75], Braun et al., [76] and Hetrick et al., [78] all indicated a small but non-significant reduction.

#### Suicide attempts

**Fig. 4:**
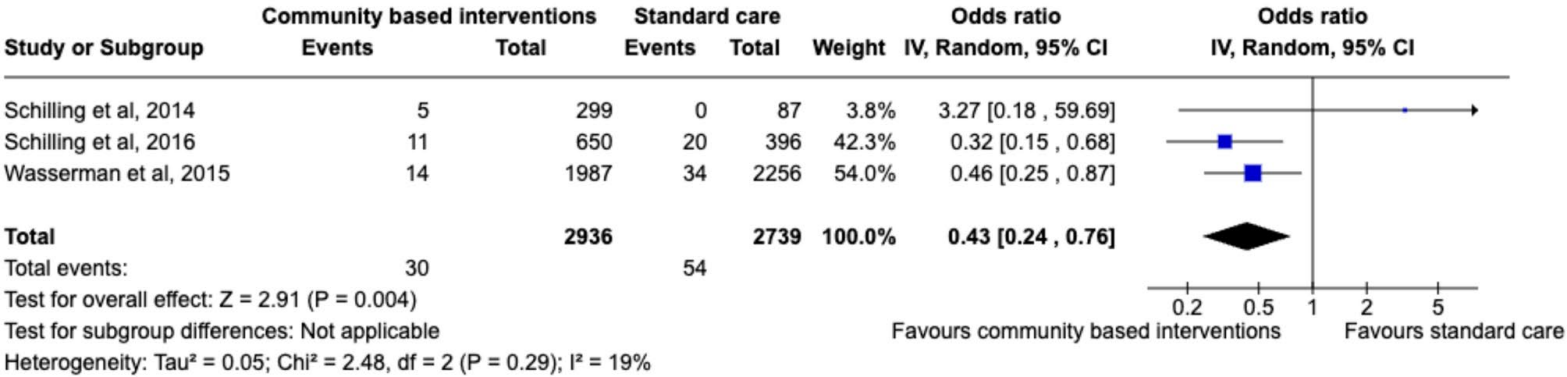
Forest plot of the efficacy of community-based intervention (versus standard care) on suicide attempt outcome

A total of six studies examined the impact of community-based programs on the reduction of suicide attempts [67, 69, 73–75, 79], three of which had sufficient data for inclusion in the meta-analysis [67, 69, 79]. The meta-analysis demonstrated a significant improvement in suicide attempts in the community-based intervention group (Pooled OR = 0.43, 95% CI: 0.24–0.76, *p* = .004) (see Fig. 4). Low heterogeneity indicated consistent effect sizes across studies (I²=19%). However, the evidence provided by the narrative synthesis was less conclusive. Three of six studies evidenced a significant reduction in suicide attempts following intervention [67, 69, 73], although only one of the three interventions examined by Wasserman et al., [69] was significant (YAM). Two further studies reported promising, but ultimately non-significant, results [75, 79]. Although Vidot et al., [75] found no significant reduction in suicide attempts in their overall sample, a significant reduction was observed specifically in adolescents with low baseline levels of parental communication. Suicide attempts were also not significantly reduced following intervention in Schilling et al., [79] when assessed as an individual outcome. However, when assessed as the composite outcome of suicidal behaviours (suicide attempt, suicidal ideation and suicide planning, collectively), Schilling et al., [79] observed a significant reduction following intervention. Kerr et al., [74] did not observe a significant reduction in suicide attempt following intervention.

#### Suicide planning

**Fig. 5:**
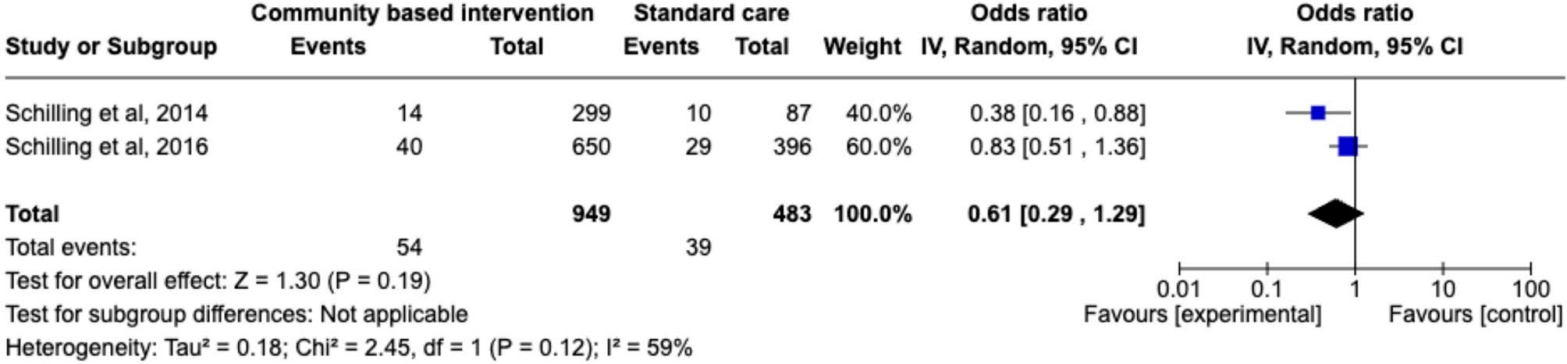
Forest plot of the efficacy of community-based intervention (versus standard care) on suicide planning outcome

Only two studies examined the effect of community-based interventions on the reduction of suicide planning [67, 79]. An exploratory meta-analysis was conducted utilising both. However, due to the low number of studies included, the reliability of the findings is limited and any conclusions drawn should be interpreted with caution. Nevertheless, the meta-analysis revealed no significant reduction in suicide planning as a result of community-based intervention (Pooled OR = 0.61, 95% CI: 0.29–1.29], *p* = .19) (see Fig. 5). The meta-analysis also revealed substantial heterogeneity (*I²*=59%). Consistent with the meta-analysis, when narratively synthesised, neither of the two studies examining suicide planning as a primary outcome evidenced a significant reduction following intervention [67, 79]. However, when suicide planning was combined with suicide attempt and suicidal ideation into a composite outcome (suicidal behaviours), a significant reduction in suicidal behaviours was observed [67, 79].

#### Non-suicidal self-injury

While studies with either STB or NSSI as a primary outcome were eligible for inclusion in this review, only one examining NSSI as a primary outcome was included [77]. Dobias et al., [77] evaluated non-suicidal self-injury (NSSI), which has been observed to predict future suicidal behaviour, as well as exist on the continuum of self-injury alongside suicide attempt [81]. Therefore, it was not excluded, although NSSI is arguably not a direct dimension of STB. Regardless, the primary outcome of non-suicidal self-injury was not significantly reduced in Dobias et al., [77], though the exploratory outcome desire to stop future non-suicidal self-injury did evidence significant, albeit short-term, reduction.

#### Quality assessment and risk of bias

Quality assessment and risk of bias for individual studies are reported in Table 3 (for RCTs) and Table 4 (for quasi-experimental studies). Of the 13 included articles, 12 were rated ‘good’ [67–70, 72–79] and one [71] was reported as fair, due to reasons such as a lack of concealment, blinding, and follow-up. As determined through GRADE criteria, the overall quality of evidence was high for studies assessing suicidal ideation, moderate for studies assessing suicide attempts, low for studies assessing NSSI and very low for studies assessing suicide planning (see Table 5). Both the suicide attempts and NSSI ratings were downgraded due to wide confidence intervals. As only one study assessed NSSI, inconsistency was not applicable, and the rating was downgraded further. Finally, the studies evaluating suicide planning featured high heterogeneity, wide confidence intervals and serious risk of bias due to their design (i.e., lack of randomisation, concealment and blinding, varied reliability of measures, as well as differences between participants at baseline).

**Table 3:** Quality assessment of randomised controlled studies using Joanna Briggs Institute (JBI) critical appraisal checklist

Author and year	Q1	Q2	Q3	Q4	Q5	Q6	Q7	Q8	Q9	Q10	Q11	Q12	Q13	Quality
Braun et al., 2023 [76]	Y	Y	Y	Y	Y	Y	U	Y	Y	Y	Y	Y	Y	Good
Dobias et al., 2021 [77]	Y	Y	Y	Y	Y	Y	Y	Y	Y	N	Y	Y	Y	Good
Grummit et al., 2022 [72]	Y	N	Y	N	N	Y	N	Y	Y	Y	Y	Y	Y	Good
Hetrick et al., 2017 [78]	Y	Y	N	U	U	Y	Y	Y	Y	N	Y	Y	Y	Good
Kerr et al., 2014 [74]	Y	Y	Y	U	Y	Y	Y	Y	Y	Y	Y	Y	Y	Good
Petrova et al., 2015 [68]	Y	U	Y	U	U	Y	U	Y	Y	N	Y	Y	Y	Good
Robinson et al., 2016 [71]	Y	U	Y	N	N	Y	U	Y	Y	U	U	Y	Y	Fair
Robinson et al., 2024 [70]	Y	U	Y	N	N	Y	N	Y	Y	Y	Y	Y	Y	Good
Sandler et al., 2016 [73]	Y	U	U	U	U	Y	Y	Y	Y	Y	Y	Y	Y	Good
Schilling et al., 2016 [67]	Y	U	N	U	U	Y	U	Y	Y	Y	Y	Y	Y	Good
Vidot et al., 2016 [75]	Y	U	Y	U	U	Y	U	Y	Y	Y	Y	Y	Y	Good
Wasserman et al., 2015 [69]	Y	U	Y	Y	U	Y	N	Y	U	Y	Y	Y	Y	Good

Y = Yes, N = No, U = Unclear; Good = Good quality (≥ 8 yeses), Fair = Fair quality (6–7 yeses), Poor = Poor quality (≤ 5 yeses)

**Table 4:** Quality assessment of quasi-experimental studies using Joanna Briggs Institute (JBI) critical appraisal checklist

Author and year	Q1	Q2	Q3	Q4	Q5	Q6	Q7	Q8	Q9	Quality
Schilling et al. [79]	Y	Y	U	Y	N	Y	U	Y	Y	Good

Y = Yes, N = No, U = Unclear; Good = Good quality (≥ 6 yeses), Fair = Fair quality (4–5 yeses), Poor = Poor quality (≤ 3 yeses)

**Table 5:** Grading of recommendations, assessment, development, and evaluation (GRADE) assessment reporting effectiveness of community-based intervention vs. standard care on outcomes of interest

Outcomes	No. of studies	Risk of bias	Inconsistency	Indirectness	Imprecision	Publication bias	GRADE quality of evidence þ
Suicidal Ideation	Twelve	Not serious	Not serious	Not serious	Not serious	Undetected	High
Suicide Attempt	Six	Not serious	Not serious	Not serious	Serious	Undetected	Moderate ‡
Suicide Planning	Two	Serious	Serious	Not serious	Serious	Undetected	Very low ‡ ¶ ^^^
Non-suicidal self-injury	One	Not serious	-*	Not serious	Serious	Undetected	Low ‡*

þ High quality: Further research is unlikely to change our confidence in the estimate of effect; Moderate quality: Further research is unlikely to have an important impact on our confidence in the estimate of effect, but still may change the estimate; Low quality: Further research is likely to have an important impact on our confidence in the estimate of effect and is likely to change the estimate; Very low quality: We are very uncertain about the estimate; ‡ Meta-analysis revealed wide confidence intervals; ¶ Moderate level of heterogeneity within results (I2 = 59%); ^^^ Studies showed notable limitations in methodological rigour; * Single study—Inconsistency not applicable; † Because of the nature of the quasi-experimental designs, risk of bias is unavoidable

## Discussion

This systematic review and meta-analysis demonstrated that community-based interventions were effective in reducing the rates of suicidal ideation and suicide attempt in CYP. However, the quantitative and qualitative analyses on if suicide planning and NSSI were reduced following intervention were constrained by an insufficient number of eligible studies, thereby making it challenging to draw confident conclusions. We suspect limited resources (such as funding constraints), organisational and methodological challenges, and a focus on clinical interventions have resulted in a paucity of community-based program evaluations eligible for this review [82–84].

Although the findings of our review suggest a positive impact, the current body of literature on the effectiveness of community-based intervention to reduce suicidal ideation remains inconclusive. For instance, a slight majority of previously published reviews and meta-analyses have reported either meaningful or promising reductions in suicidal ideation post-intervention [40–42, 44, 85–91]. In contrast, a considerable number of reviews reported little to no evidence of reduction of suicidal ideation following an intervention, due to reasons such as a scarcity of studies eligible for inclusion, and methodological weaknesses of the studies which were included [24, 43, 92–94]. However, many previous reviews with results both consistent and inconsistent with ours reported a lack of methodological rigour in their included studies, such as unclear, moderate or high risk of bias [89, 94], not conducting risk of bias assessments [41], statistical imprecision [42], no evidence of replicability in included studies [92], a lack of standardised outcome measures [88], the use of single-item measures [42], and insufficient follow-ups conducted by included studies [43, 93]. Although our review rated the studies assessing suicidal ideation as of high quality, short follow-up durations (under 6 months) [67, 68, 76, 77, 79] and high attrition rates [67, 70, 77] were key limitations. Notably, previous meta-analyses with similar scopes have also reported short follow-up durations and variable attrition rates as prevalent limitations among school-based interventions [90, 95]. Additionally, a number of previous reviews assessing a broader range of program varieties (e.g., community-based alongside clinical and pharmacological) reported a lack of community-based programs eligible for inclusion, decreasing reliable conclusions regarding the efficacy of these interventions in reducing suicidal ideation [45–47, 93]. Taken together, the current body of literature is undermined by pervasive methodological issues. The variation in study quality may explain the lack of consensus across reviews, including why our review found significant results where others did not. Another possible explanation may be differences in the inclusion criteria across reviews. Our review focused on interventions in any community-setting (including, but not restricted to, school-settings), where the target was reduction in STB in CYP. In contrast, the reviews which report little to no evidence that community-based interventions reduce STB in CYP typically adopted a broader scope than ours. This includes assessing clinical and pharmacological interventions alongside community based interventions [24, 43, 93], combining the results of participants under and over 18 years [43], evaluating outcomes beyond STB (such as depression, wellbeing or attitudes towards suicide) [93, 94] and including a wider range of study designs, including interventions without a control [93]. The stringent scope adopted by this review, particularly RCTs and controlled studies, may explain why we observed significant results not found in other reviews.

Consistent with other relevant reviews focusing on young people, we observed that community-based interventions had either a meaningful or tentative effect on reducing suicide attempts [40, 41, 43, 45, 85–88, 92, 93, 96]. However, some of these reviews also report limitations, such as weak methodology of included studies [85, 92, 96], a lack of community-based interventions to include [45], or that the included evaluations more commonly assessed outcomes other than STB [93]. Alternatively, two previous reviews reported contrasting results [24, 47]. Zalsman et al., [24] observed significant effectiveness of school-based interventions only, but not for other community-based interventions, and York et al., [47], reported insufficient evidence supporting the reduction of attempts following intervention. Liljedahl et al., [96], report that school-based interventions specifically do, albeit tentatively, reduce suicide attempts in CYP, while also highlighting the lack of high-quality evidence available. Similarly, past reviews and studies focused on adult populations also reported promising but uncertain results, primarily due to methodological weaknesses of the included interventions [84, 97–99]. Overall, this suggests that variation in findings across the literature may be a result of the mixed-quality interventions included in these reviews. In order to confidently and meaningfully fill the gap in community-based programs for the reduction of suicide attempt among CYP, high-quality research is critically needed.

In this review, findings of the suicide planning outcome was compromised by moderate heterogeneity and limited studies, so any conclusions drawn should be done with utmost caution. Moreover, the qualitative synthesis revealed that community-based interventions did not effectively reduce suicide planning when assessed independently, but did when enveloping planning into the composite outcome of suicidal behaviours. Aligned with our findings, Zalsman et al., [24], in their comprehensive review on suicide prevention strategies (including community-based) from 2005 to 2014, reported no evidence to suggest community-based programs were effective in reducing suicidal behaviours. However, in contrast, three reviews published since 2014 did report a decrease in suicidal behaviours (including suicide planning), suggesting recently developed interventions may be more effective [41, 42, 44]. Additionally, the single study included in this review reporting on NSSI observed no significant reduction in non-suicidal NSSI following intervention (Project SAVE [Stop Adolescent Violence Everywhere]) [77]. However, Dobias et al., [77] also reported high attrition rate (with only 42% of participants completing the 3-month follow-up) and potential data limitations due to the retrospective self-report format of measures utilised. Contrary to this non-significant result, other reviews assessing the reduction of NSSI following community-based programs mostly indicated meaningful or promising effects [41–44]. This may be attributed to outcome construction: these reviews grouped NSSI into composite categories (e.g., “suicidal behaviours”,) potentially obscuring differential effects when NSSI is analysed independently.

The current systemic review has several strengths and limitations. The inclusion of the meta-analysis synthesises results from the diverse range of programs included, revealing a more robust understanding of the overall effectiveness. That 12 of 13 studies included achieved a rating of ‘good’ internal validity, with the thirteenth rated as ‘fair’, further strengthens confidence in the meta-analysis results. Additionally, previous reviews with broader outcome ranges report that the lack of outcome standardisation makes drawing confident conclusions challenging [47, 92]. Our review addresses this by only including studies with a primary outcome of STB or NSSI. Both the standardisation of outcomes and meta-analysis are particularly important given the variation in outcomes, target strategies (i.e., universal, selective, indicated and upstream), modalities, and intervention techniques present in the literature. This clarifies the uncertain results of the narrative synthesis and provides a foundation on which to build further research, which is critically needed. In keeping with our inclusion criteria, our review is also distinctly positioned within the field, though the strict focus has yielded a smaller pool of eligible school/community trials. Limiting data collection to pre-specified primary STB/NSSI endpoints improves construct validity, reduces selective-reporting bias, and enhances comparability across studies [49, 57, 59]. This approach aligns with guidance emphasising that primary outcomes drive power and interpretation, whereas secondary outcomes are typically supportive/exploratory and at higher risk of biased or imprecise estimates [58, 60]. Our review is distinct to past reviews as they have assessed adults alongside children without separation [43, 45], either restricted their assessment of community-based interventions to those in school-settings only [40–42, 92, 93] or broadened the scope to include clinical and pharmacological interventions as well [24, 44], reviewed studies which evaluated STB as a secondary outcome, or evaluated related but tangential outcomes (e.g., attitudes towards suicide) [43, 44].

However, there are several limitations which restrict confidence in any conclusions drawn regarding the effectiveness of community-based interventions in reducing suicide planning and NSSI. This includes that only two eligible studies including suicide planning as an independent outcome were identified [67, 79], both of which evaluated the Signs of Suicide program. The moderate heterogeneity further complicates interpreting the results. Observed heterogeneity and interpretive uncertainty may partly reflect differences in how included studied defined and measured STB outcomes and NSSI. Sensitivity analyses were not run on the outcomes of suicide attempt or suicide planning, given the already limited number of studies in each meta-analysis. Therefore, the robustness of these findings may be affected and must be interpreted with caution. Furthermore, short follow-up windows and variable attrition rates were key limitations of the included studies, introducing additional uncertainty in our results. Three of the included studies either did not follow-up [68, 71] or had a follow-up duration under 3-months [76], four more followed-up at 3-months post-intervention only [67, 77–79], and four reported high attrition rates [67, 70, 77, 79]. These features may bias pooled effects in either direction: short windows can either inflate early gains or miss delayed change, while differential attrition can either attenuate or exaggerate effects depending on who is lost to follow-up. We, therefore, interpret effect estimates cautiously and reflect these issues in our certainty assessments. In addition, the single, non-significant study on NSSI received a GRADE rating of low [77], while suicide planning received a rating of ‘very low’. Penultimately, generalisability of these results is restricted as all of the included interventions were completed in high-income countries. Finally, the true prevalence of STB is likely underestimated in the included studies due to underreporting and misclassification in clinical, administrative, and survey contexts [100], which can translate into biased or imprecise effect estimates.

We limited inclusion to 2013–2024 to capture the modern definitional consensus (CDC uniform definitions [49]; DSM-5 definitions [51] and widespread use of standardised measures, e.g., C-SSRS [54, 55]). Nevertheless, measurement still varies: the same individuals report ideation/attempts differently across assessment modes, with the highest disclosure on anonymous surveys [101]. Furthermore, small wording changes can shift ideation/planning prevalence [102]. Even widely used tools like the C-SSRS have practical psychometric limitations in real-world clinical settings (e.g., emergency departments), including low predictive value/sensitivity for near-term suicide [103–105]. Cross-national terminology (e.g., the umbrella term of “self-harm” versus intent-based NSSI/attempt) differs. These factors likely contributed to inter-study heterogeneity (particularly for planning and NSSI) and should temper interpretation of pooled effects. Future studies should pre-register intent-based outcome definitions (ideation, plan, attempt, NSSI), use validated multi-item measures or standardised interviews, report item wording verbatim, and include longer follow-up while minimising attrition.

### Implications for practice and policy

The findings from this review suggest significant implications for both practice and policy in addressing suicidal ideation and suicide attempts through community-based interventions. These results advocate for the widespread implementation of community-based programs. However, the variability in outcomes across different interventions highlights the need for targeted, tailored approaches. From a policy perspective, these findings support the integration of community-based suicide prevention programs into national mental health strategies. Given the promising results of this review, specifically with regards to suicidal ideation and suicide attempts, continued research in community-based approaches is warranted, provided the research adheres to strong methodological rigour. Researchers should continue to build on this body of evidence to shape future implications, as well as better support its integration into practice and policy.

### Directions for future research

New interventions may provide direction for future research. There is a growing advocacy for research on targeted interventions aimed at those experiencing immediate distress or crisis, such as community helplines and outreach services. For example, in Australia, Lifeline helpline, Suicide Prevention Outreach Teams (SPOT) and Safe Haven offer accessible support and have shown to reduce both attempted suicides, and suicide deaths, by connecting individuals with peers, community members and clinicians who de-escalate and empathise [100, 106–108]. Another avenue for future research is the development and assessment of programs utilising a digital or semi-digital modality, either standalone or to augment in-person interventions. Digital and semi-digital interventions have the potential to be valuable tools in addressing STB in youth, offering increased accessibility and scalability [109, 110], flexibility [111] and lower dropout rates compared to face-to-face care [112]. Digital interventions may also uniquely offer anonymity, which may increase reporting and offer a closer estimate of the true prevalence of suicidal thoughts and behaviours [102, 113]. Digital interventions are also showing applicability and effectiveness in reaching CYP in LMICs [114], although these results have not yet been assessed with STB specifically. Finally, and perhaps most crucially, future research should consider developing interventions for vulnerable but underrepresented populations to ensure people are able to access the support they need. This includes those with developmental disabilities such as Autism Spectrum Disorder, Indigenous peoples, LGBTQ + individuals, and rural communities, all of whom are at significantly higher-risk for suicidal thoughts and behaviours [115–118]. These interventions should be tailored, targeting the risk-factors specific for each population group, as the benefits identified through general populations may not be transferrable. Also warranting the development of context-specific, culturally and socially sensitive programs are CYP from LMICs, who face a lack of mental health programs disproportionate to their rates of STB and NSSI [7, 9, 10, 119]. Co-designing interventions with these populations may be one method of improving relevance and addressing the specific challenges faced [120]. Overall, there are a myriad of potential directions for future research, all of which may aid policymakers and practitioners in the implementation and integration of effective, high-quality programs in the future.

## Conclusion

Our systematic review and meta-analysis revealed that community-based mental health programs significantly reduce suicidal ideation and suicide attempt in children and young people. However, it is challenging to answer whether community-based mental health programs are able to effectively reduce suicide planning or NSSI in this population. Both of these outcomes were constrained by a paucity of evaluations eligible for inclusion. A lack of studies assessing STB directly was observed to be a trend across the current body of literature, as was weak methodological rigour. Therefore, future research should prioritise improving the quality and breadth of intervention evaluations. Further recommendations for future research include interventions targeting those in immediate distress, those from vulnerable and underrepresented groups, and finally, to explore and advance digital interventions.

## Data Availability

All data supporting the findings of this study are available within the paper.

## Abbreviations

CIs: Confidence intervals
CYP: Children and young people
ED: Emergency department
FBP: Family Bereavement Program
GRADE: Grading of Recommendations Assessment, Development and Evaluation
HICs: High-income countries
LICs: Low-income countries
NSSI: Non-suicidal self-injury
OR: Odds ratios
Project SAVE: Project Stop Adolescent Violence Everywhere
QPR: Question, Persuade, and Refer
RCT: Randomised Controlled Trial
RevMan: Review Manager
SPOT: Suicide Prevention Outreach Teams
STB: Suicidal thoughts and behaviours
